# From powders to bulk metallic glass composites

**DOI:** 10.1038/s41598-017-06424-4

**Published:** 2017-07-27

**Authors:** Lisa Krämer, Yannick Champion, Reinhard Pippan

**Affiliations:** 1 Erich Schmid Institute of Materials Science, Austrian Academy of Sciences, Leoben, Austria; 2 Univ. Grenoble Alpes, CNRS, Grenoble INP, SIMaP, F-38000 Grenoble, France

## Abstract

One way to adjust the properties of materials is by changing its microstructure. This concept is not easily applicable on bulk metallic glasses (BMGs), because they do not consist of grains or different phases and so their microstructure is very homogeneous. One obvious way to integrate inhomogeneities is to produce bulk metallic glass composites (BMGCs). Here we show how to generate BMGCs via high-pressure torsion (HPT) starting from powders (amorphous Zr-MG and crystalline Cu). Using this approach, the composition can be varied and by changing the applied shear strains, the refinement of the microstructure is adjustable. This process permits to produce amorphous/crystalline composites where the scale of the phases can be varied from the micro- to the nanometer regime. Even mixing of the two phases and the generation of new metallic glasses can be achieved. The refinement of microstructure increases the hardness and a hardness higher than the initial BMG can be obtained.

## Introduction

Bulk metallic glasses (BMGs) have advantages over crystalline metals including high hardness, high elastic energy storage and high corrosion resistivity, but have also some major drawbacks. BMGs are relatively brittle and especially show poor ductility in tensile testing^1^. Changing the microstructure is a common way to tune properties of materials. It was shown for BMGs that changing the local short range order through rejuvenation by thermal or mechanical cycling will influence mechanical properties^2–4^. Another way is to produce composites containing an additional amorphous or crystalline phase^5^. One prominent route is to partly crystallize the amorphous sample by either choosing a slower cooling rate, another composition or reheating the BMG up to the crystallization temperature^6–11^. Other routes are casting the BMG with crystalline parts as springs, tubes, particles or wires^12–17^ or using warm extrusion of a mixture of powders^18^. Drawbacks of these strategies are the inhomogeneous microstructure and sometimes the brittle crystalline phase^19, 20^. Another idea is to use powder metallurgy and sintering to fabricate bulk metallic glass composites (BMGCs) and despite problems with porosity, promising results have been published^21–23^. For producing crystalline composites, also severe plastic deformation (SPD) has been used in the past^24, 25^. Even though many different SPD techniques (equal channel angular extrusion, accumulative roll bonding and many others) have been developed, high pressure torsion (HPT) is used for this work because it has some major advantages especially for research. The applied strain can be easily varied by changing the number of rotations; many commonly brittle materials can be deformed due to its high nearly hydrostatic pressure and even powders are possible as initial material. By using powders, a wider range of compositions become feasible compared to conventional casting. Super saturated solid solutions are producible and unfavorable phases can be avoided^26–30^. HPT was already used on MGs, on one hand to change the properties of BMGs by structural rejuvenation^3, 4, 31–34^ and on the other hand to fabricate BMGs and BMGCs^35–44^. Zr-MG powder was compacted and deformed via HPT beforehand. It could be shown that fully dense and amorphous specimens without cracks and pores can be fabricated with sufficient applied strains and no crystallization occurred during the HPT process^45^.

The aim of this study is to show that new types of BMGCs can be produced via SPD. Questions addressed are: what are the obtainable limits of the metal-metallic glass composites in scale and content, and how far can we extend the field of bulk metallic glasses. The initial materials are powders (Zr-MG and crystalline Cu) that were mixed and then consolidated, welded together and refined by HPT. Four different compositions (Zr-MG Xwt% Cu, X = 20, 40, 60, 80) were produced as well as single phase Zr-MG samples as reference. To investigate the influence of the degree of deformation and the ratio of the two phases on the evolution of the microstructure and mechanical properties, scanning electron microscopy (SEM), X-ray diffraction (XRD) and hardness measurements were used.

## Microstructural evolution as a function of strain

**Figure 1:**
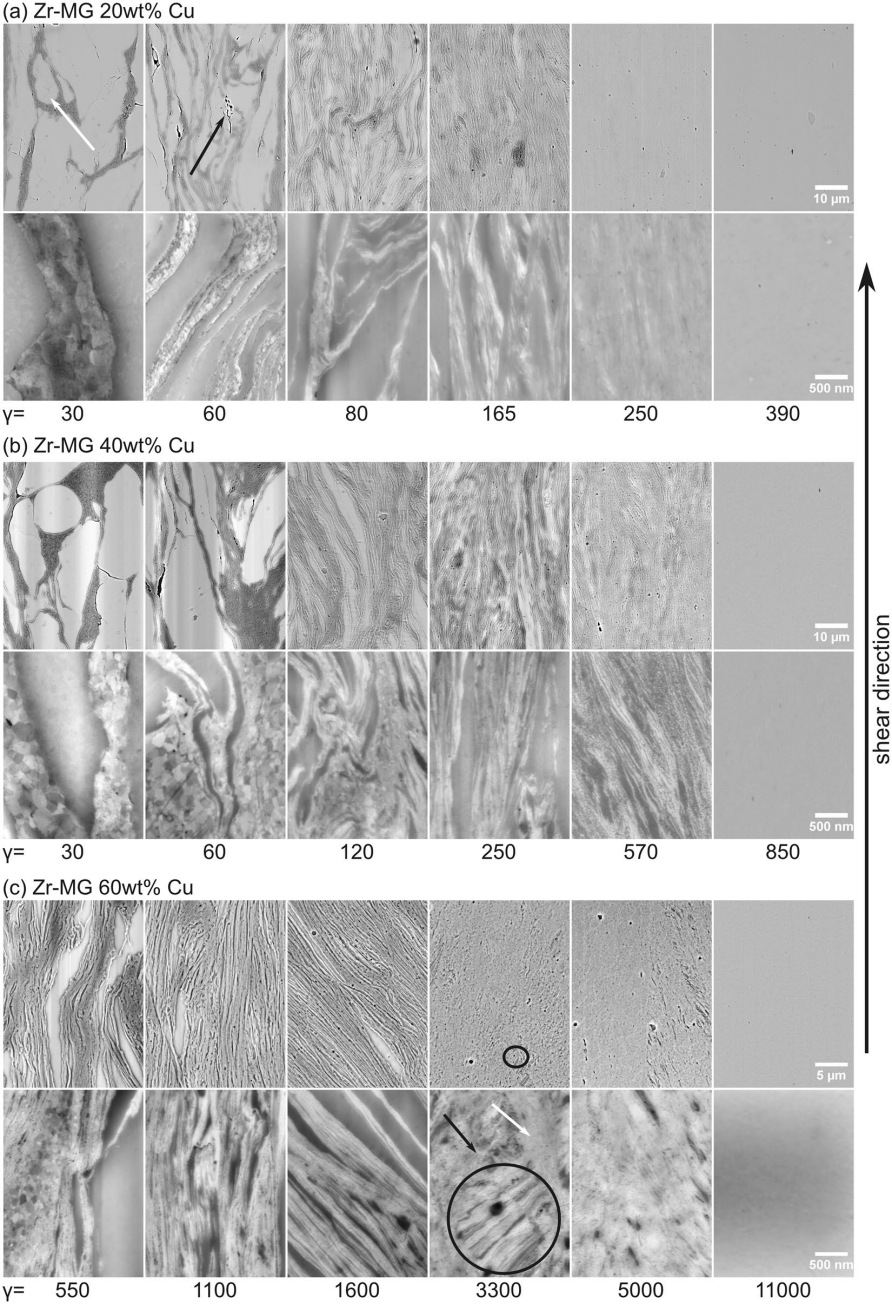
Illustration of SEM micrographs of (**a**) Zr-MG 20 wt% Cu, (**b**) Zr-MG 40 wt% Cu, and (**c**) Zr-MG 60 wt% Cu. The top and bottom row of micrographs are taken at different magnifications. The evolution of the microstructure can be seen in dependence of the applied strain. The deformation increases from the left to the right and the microstructure changes from Zr-MG particles glued together by crystalline Cu to a lamellar structure. These lamellae refine with increasing applied strain until a complete mixing of the two phases occurs. Take notice in the increase of applied strain at higher contents of Cu.

In Fig. 1a, the microstructure of Zr-MG + 20 wt% Cu as a function of the applied strain is presented (the shear direction is indicated with an arrow). All micrographs are backscatter-detector images to distinguish easily between Cu and the Zr-based MG. Images in the lower row have a significant higher magnification. The two phases in the coarse state differ in their mechanical properties, which strongly influences the deformation. In the beginning, Cu sustains most of the deformation as it has a lower hardness and yield strength. Zr-MG is more difficult to deform and the initial particles are easily distinguished as they change their initial shape and size only marginally (one of those particle is marked with a white arrow in Fig. 1a). Cu works as a glue and holds the amorphous particles together. Due to the heavy plastic deformation, its grain size is refined down to the range of several hundreds of nanometers. Higher strains (see micrographs for γ = 50) force the Zr-MG to deform and bond together. Therefore, the amorphous phase forms long elongated regions with a length of about 100 µm in the shear direction and widths up to 10 µm, where some features from the initial particles are still detectable (area marked by the black arrow). The Cu bands become thinner and more evenly distributed, but large Cu-containing areas can still be found. The Cu grains refine and become less than 100 nm in size. Micrographs at γ = 80 show a further refinement of the microstructure. Cu and Zr-MG lamellae decrease in length and in width: All of the Cu lamellas are thinner than 1 µm and most of them exhibit a thickness of 20 nm. Cu grains are not detectable in the SEM micrographs anymore, but the thickness of the thinner lamellas can be seen as an upper limit for the grain size in this bands. The elongated regions of the amorphous phase shrinks below 100 µm in length and 10 µm in width. From γ = 80 to γ = 165 a pronounced increase of the number of the thin Zr-MG lamellas occurs, so that the Cu network in the images at low magnification, is in reality a Cu rich region consisting of Cu lamellas with lengths below 1 µm and widths below 100 nm separated by about equally sized amorphous lamellas. Additionally, the boundaries between the two phases change from sharp and easily detectable to more blurry interfaces as the Cu starts to mix with the amorphous phase at higher strains. This process starts already at γ = 80, but is clearly recognizable at γ = 165. The mixing of the two phases progresses with increasing applied deformation. At γ = 250 elongated Cu rich and Zr-MG rich regions can still be detected with smooth transitions in between, but at γ = 390 Cu the SEM micrographs seems to show a single phase metallic glass.

The ratio of the amorphous and crystalline phase can be easily modified by varying the ratio of the two initial powders. To investigate the influence of the ratio of the two phases, compositions with higher contents of Cu were produced and SEM micrographs of the cross sections of samples with 40 wt% Cu at different applied strains are shown in Fig. 1b. At first glance the major difference visible in the composition with less Cu in Fig. 1a, is the higher amount of the crystalline phase, but the deformation mechanism does not differ. The softer Cu phase starts to deform first, but with higher amount of applied strain, the harder Zr-MG starts to form long elongated bands, which shrink with increasing deformation. At higher strains, the two phases start to mix again until a homogeneous material is formed. However, by comparing the micrographs for the two compositions, it can be clearly seen that a higher content of Cu requires a higher strains to reach the same refinement of the microstructure. To obtain the saturation microstructure (i.e. the monolithic metallic glass), the applied strain for Zr-MG + 40 wt% Cu has to be doubled compared to the Zr-MG + 20 wt% Cu.

The content of Cu was further increased to investigate the limits of mixing and to see any resulting change of the deformation behavior. In Fig. 1c, SEM micrographs of Zr-MG 60 wt% Cu can be seen. Compared to the samples with 20 and 40 wt% Cu, the deformation behavior in the beginning does not change and a lamellar structure is formed (see γ = 550–1600). At higher applied strains (γ = 3300–5000), the micrographs exhibits a deviation from the deformation behavior from the samples with low Cu content. The lamellas shrink in thickness with higher applied strain, but mainly a break-down of the lamellar configuration occurs. A transition microstructure develops with residual lamellar blocks embedded (see circled areas in Fig. 1c) in a nanocrystalline (see black arrow) and amorphous structure (see white arrow). At γ = 11000, both phases seem to be fully mixed and a single phase microstructure is obtained. The amount of strain needed to reach this saturation microstructure is about 10 times higher than for Zr-MG 40 wt% Cu and about 20 times higher than for Zr-MG 20 wt% Cu.

**Figure 2:**
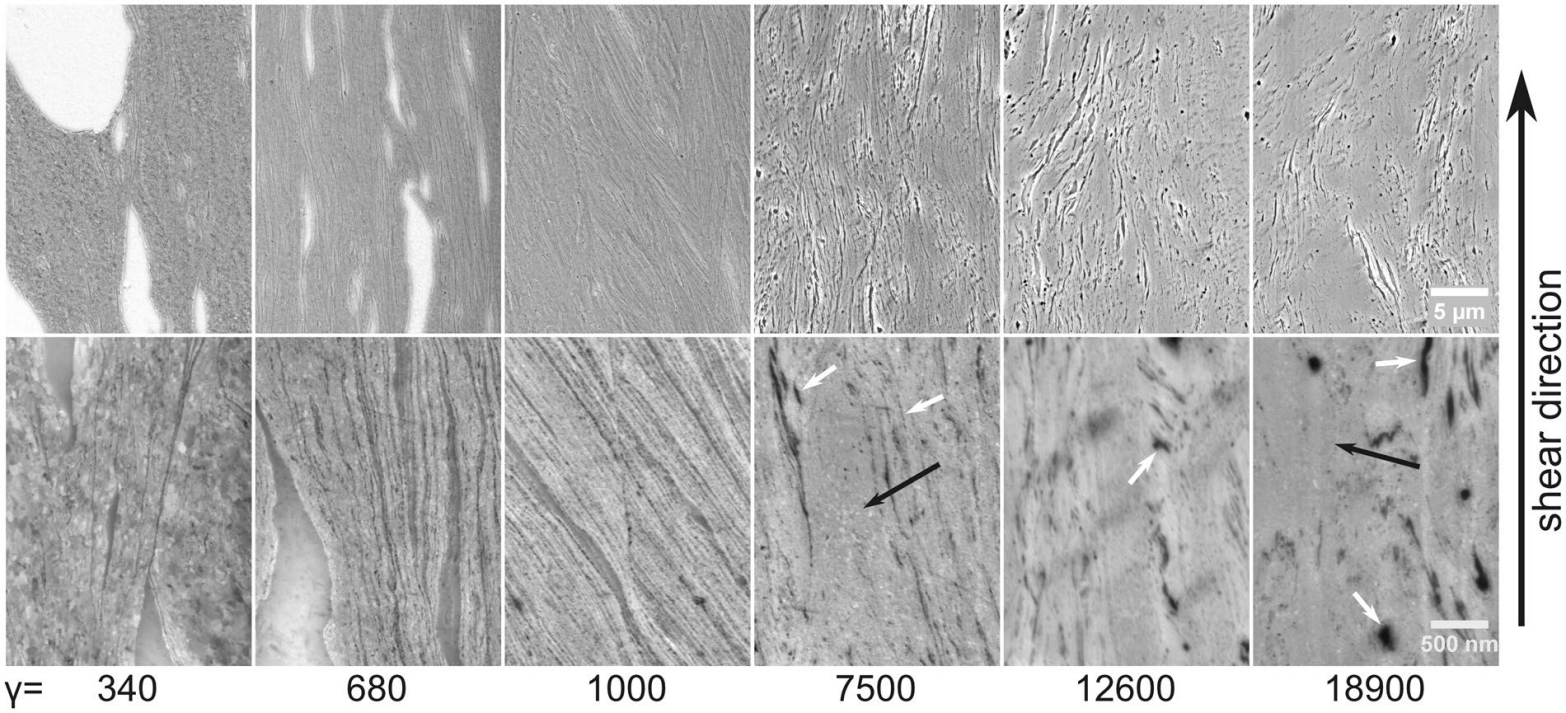
Illustration of SEM micrographs of Zr-MG 80 wt% Cu. The amount of the softer phase (Cu) is too high to fully mix the two phases and Zr-MG bands and crystalline Cu rich regions characterize the microstructure. Even after γ = 18900, the microstructure does not significantly change and it is assumed that a saturation is reached.

The composition with the highest Cu content investigated was Zr-MG 80 wt% Cu. SEM micrographs of the cross sections are displayed in Fig. 2. Again, the Zr-MG particles elongate and a lamellar structure is obtained in the beginning, but at an applied strain of γ = 5000 and higher, the lamellas start to break and the microstructure does not change significantly anymore even after applying extremely high strains (up to γ = 18900). Since the microstructure does not change significantly even after this enormous increase of the applied strain, it is assumed that a saturation is reached. The saturation microstructure seems to consist of a nanocrystalline Cu-rich matrix (in Fig. 2 at higher magnification crystals indicated with black arrows can be seen as small freckles in the light grey area) in which elongated Zr-MG bands (some are indicated with a white arrow in Fig. 2) with a length of several micrometers are embedded.

**Figure 3:**
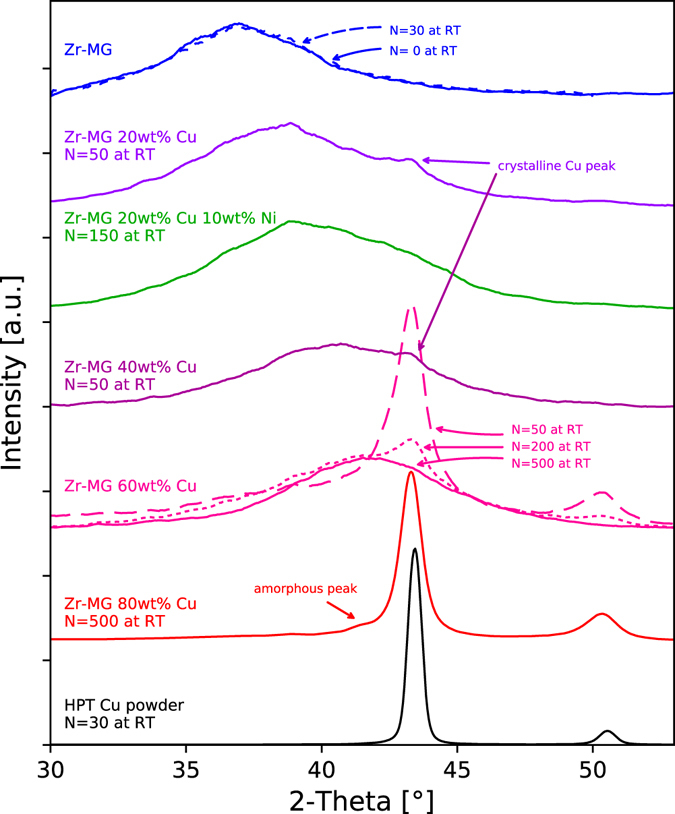
XRD results of different composite concentrations. After sufficient deformation, all compositions except Zr-MG 80 wt% Cu show one broad amorphous peak. For Zr-MG 60 wt% Cu, the evolution of the peaks with different numbers of rotations is indicated: The dominant Cu peak shrinks with increasing strains until only the amorphous peak remains after 500 rotations. The amorphous peak shifts to larger angles with higher Cu content.

The evolution of the microstructure is reflected also in XRD measurements. XRD profiles of samples from Zr-MG (undeformed and deformed), crystalline Cu, and the four compositions investigated in a near saturated condition are displayed in Fig. 3. The measured range is concentrated on the first (and strongest) peak of Zr-MG, which is very broad and has low intensity compared to the more distinctive peaks of crystalline Cu. The XRD results of a HPT deformed pure Cu powder are also depicted in Fig. 3, which has a grain size of about 100 nm^24^. The XRD peaks of the undeformed and deformed Zr-MG coincide exactly, which indicates that the HPT process does not have an influence on the single phase BMG. The influence of the applied strain can be clearly seen at the three samples of Zr-MG 60 wt% Cu as the number of rotations in the HPT process was varied from 50 to 200 and 500. The two Cu-peaks are very dominant for the specimen with 50 rotations, but shrink as the applied strain increases. After 200 rotations, the first Cu peak has a similar height as the amorphous peak and both Cu peaks broadened significantly, which can be caused by two effects: smaller crystal sizes and higher defect densities in the crystals. The crystalline peaks disappear after 500 rotations and only the amorphous peak remains.

For the other Cu concentrations, only one sample with a microstructure near the saturation is shown. This saturation is reached after different degrees of deformation. For Zr-MG 40 wt% Cu and Zr-MG 20 wt% Cu, 50 rotations are nearly sufficient to achieve a fully amorphous sample and only a minor peak indicates remaining crystalline Cu (indicated with arrows in Fig. 3). On the other hand, even after 500 rotations Zr-MG 80 wt% Cu shows a dominant Cu peak and a very weak amorphous peak (indicated with an arrow in Fig. 3). Comparing the crystalline peak of Zr-MG 60 wt% Cu and Zr-MG 80 wt% Cu to the pure Cu sample, the Cu peak is shifted to lower angles due to partial solution (or formation of a supersaturated solid solution) and broadens mainly due to the small crystalline size. Furthermore, mixing Cu into the amorphous phase shifts its peak positions to higher 2-Theta values and the difference between Zr-MG and Zr-MG 60 wt% Cu is approximately 5°. In order to demonstrate the possibility of analyzing the alloying region for generation of new metallic glasses, a sample with Zr-MG 20 wt% Cu 10 wt% Ni was investigated and after 150 rotations a fully amorphous structure is obtained.

## Effect of strain on the mechanical properties

**Figure 4:**
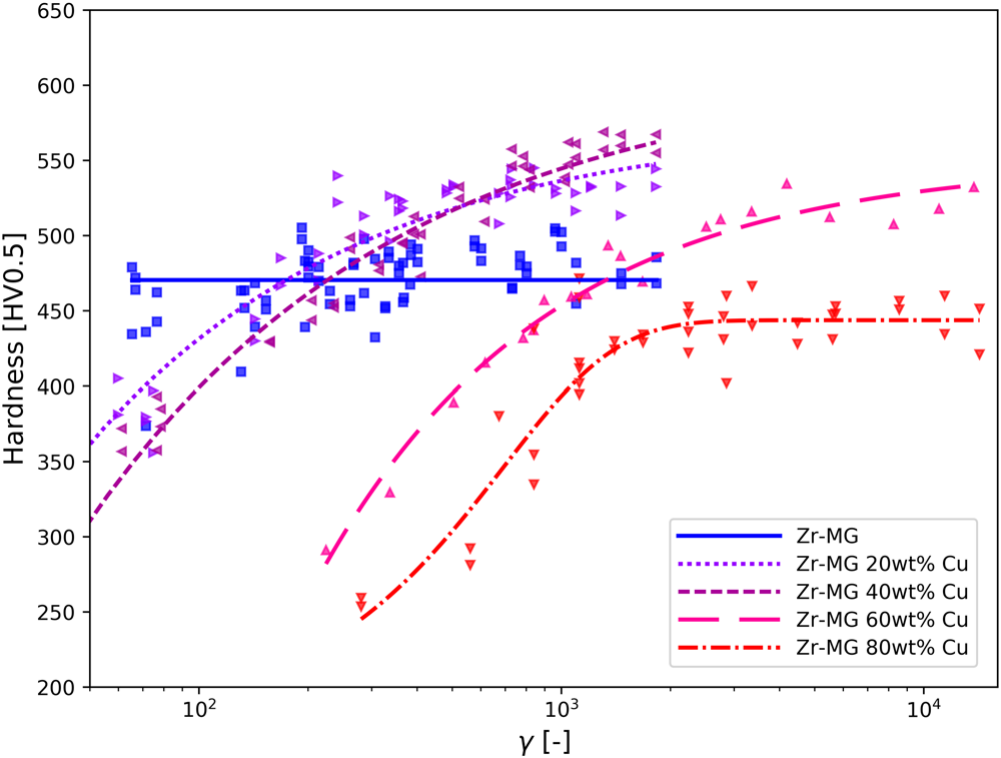
Illustration of Vicker’s hardness evolution as a function of the shear strain of all compositions and Zr-BMG. The hardness of Zr-MG is constant independently of the applied strain, but the hardness of all compositions increases with increasing strains. In the case of Zr-MG 80 wt% Cu, the hardness does not reach the hardness of Zr-MG, but all other compositions become harder than the two initial materials. The shape of the curve depends on the composition, higher contents of Cu shifts the curve to higher strains.

**Figure 5:**
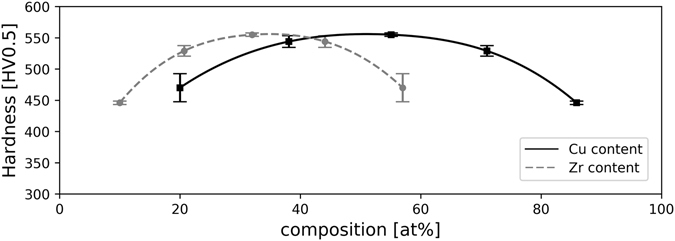
Approximated hardness at saturation for all investigated compositions. The highest hardness can be found for the sample with 40 wt% Cu (32at% Zr and 55.1at% Cu).

Microstructure influences strongly ﻿the mechanical properties, which were investigated in this study by using hardness measurement. In Fig. 4, the Vicker’s hardness is plotted versus the applied strain (see methods). Since the strain varies over about three orders of magnitudes, several specimens have been used. The fitted lines are for guiding the eye. The hardness of the single phase Zr-MG scatters strongly at low applied strain, because the particles are not welded together completely in this early state of deformation. To eliminate an effect from the HPT deformation on the hardness beside consolidation, undeformed powder and a Zr-MG sample with 30 rotations were investigated via nanoindentation. Both conditions show the same hardness with 6.12 ± 0.33 GPa for the powder and 6.11 ± 0.07 GPa for the deformed specimen. The influence of the applied strain is more pronounced for the BMGCs, because not only the particles are welded together but also a refining and mixing of the two phases occur. Three compositions (with 20, 40 and 60 wt% Cu) show a similar behavior. At low strains, the hardness is lower than the single phase Zr-MG but increases strongly with deformation. The slope decreases with higher deformation and the curves level off at a hardness higher than the Zr-MG. Exceeding the hardness of both initial powders can only be explained by mixing the two phases, shifting the chemical composition and so forming a new metallic glass with higher hardness. Higher contents of Cu shift the curve to the right, which means that welding, refining and mixing require more strain if the fraction of the softer phase is higher. This behavior of the hardness corresponds to the evolution of the microstructure where the influence of Cu can also be seen clearly by comparing micrographs of the different composition. Increasing the content of Cu in the amorphous phase also leads to higher hardness (see Zr-MG 20 wt% Cu and Zr-MG 40 wt% Cu), but it requires more deformation. Zr-MG 80 wt% Cu also show an increase in hardness with larger applied strains, but the slope is less steep compared to the other compositions and the hardness of Zr-MG is not reached even after γ = 10^4^. The material gets harder, but the rate is so slow that a real mixing (as it is the case for the other three compositions) is not practical, as days are needed to achieve sufficient deformation. Not only the shape and position of the hardness curves depend on the composition, but also the hardness at saturation. In Fig. 5, the approximated hardness for the saturated microstructure depending on the composition is shown and the highest value is reached for the composition with 40 wt% Cu (which corresponds to 32at% Zr and 55.1at% Cu). The hardness drops by approximately 15% for Zr-MG compared to Zr-MG 40 wt% Cu.

## Discussion

**Figure 6:**
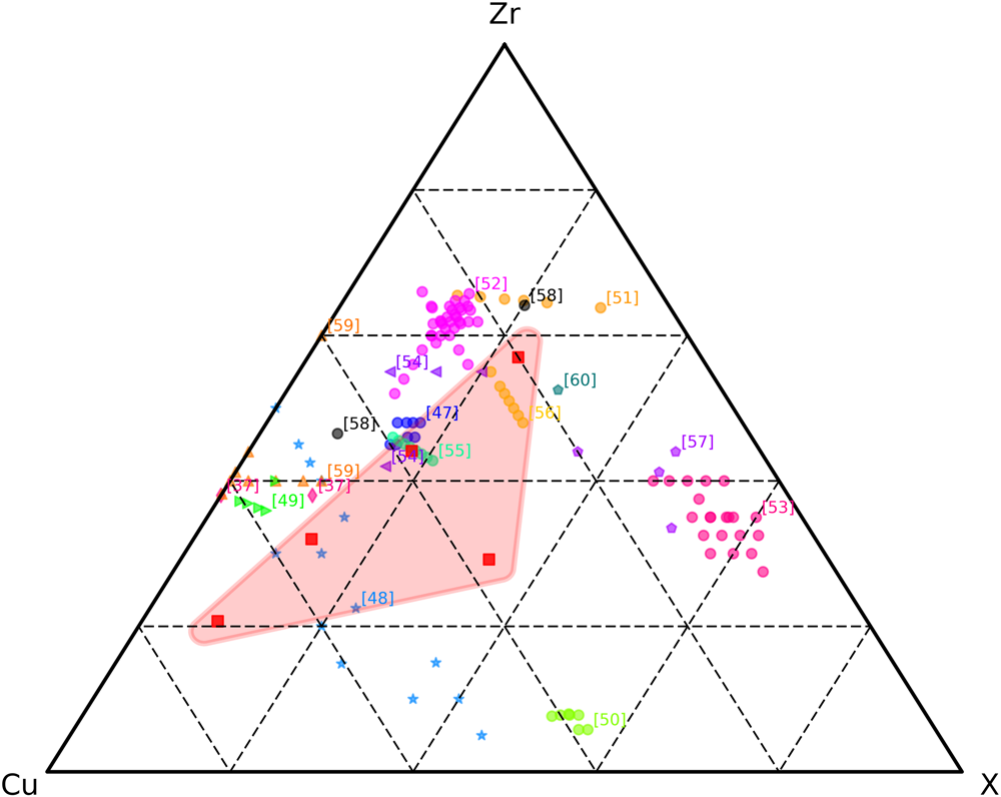
Chemical composition of BMGs from this study and literature are shown^37, 47–60^. All elements except Cu and Zr are summed up in X to enable a comparison. In this study, the chemical composition is changed by dissolving Cu into the Zr-MG and the range is marked. An additional composition with Zr-MG 20 wt% Cu 10 wt% Ni is added to show the extensive range of possible compositions.

The microstructure of the HPT deformed Cu + Zr-MG mixture and their mechanical properties depend strongly on the composition and on the applied strain. At low strains, a bulk composite is formed, in which the elongated phases start to refine and the hardness increases strongly. Increasing the applied strain, continuous thinning of the lamellae can be found for 20 and 40 wt% of Cu, whereas, the lamellas tend to break down at higher contents of Cu. For 60 and 80 wt% Cu, the length of the lamellae decreases more than their thickness. Additional, a mixing of the two phases takes place and at very high strains, a single phase BMG is obtained for all composition except Zr-MG 80 wt% Cu. However, compared to the beginning the microstructure changes slower as the applied strain increases, which is also reflected in the hardness curves as all level off at higher degrees of deformation. The strain necessary to reach saturation increases significantly with increasing content of the softer crystalline phase (e.g.: more than 20 times if the content of Cu is increased from 20 wt% to 60 wt%). This can be seen in SEM, XRD and the hardness measurements (more Cu also shifts the hardness curves to higher strains). The position of the amorphous peak shifts to higher degrees as more Cu is mixed into the MG (the peak position of Zr-MG and Zr-MG 60 wt% Cu differs with approximately 5°), but it can also been shown how the crystalline Cu in the composites is affected. As the applied strain increases, the crystalline peak broadens due to grain refinement. The grain size becomes smaller during HPT deformation in the presence of a second phase than in a single phase crystalline material. Mixing of the two phases takes place also for Zr-MG 80 wt% Cu. In this case, a part of Zr-MG is dissolved in the crystalline Cu, it remains crystalline (nanocrystalline) and a supersaturated solid solution is formed. The exact quantity is hard to obtain, because the Zr-MG contains four other elements beside Cu (if only Zr was considered, the peak shift would indicate dissolving 0.16 at% into the crystalline Cu^46^). Two effects could simultaneously prevent a full mixing of the two phases for this composition: The solubility of Cu in the amorphous phase is restricted and the limit lies between 71at% (Zr-MG 60 wt% Cu) and 85.9at% (Zr-MG 80 wt% Cu). Secondly, the amorphous particles are not forced to deform as strongly as in the other compositions, because the high content of soft Cu carries all of the deformation. The consequences of the second effect is the possibility of a further mixing of the two phases if the applied strain is pushed to higher values. However, this can only happen if the emerging amorphous phase has a higher strength and forces the initial MG to deform further. Comparing the approximated hardness at saturation, it can be seen that all compositions show a higher hardening than Zr-MG 80 wt% Cu. The highest value is obtained for Zr-MG 40 wt% Cu. Zr-MG 80 wt% Cu still consists considerably of a crystalline phase but has already a similar hardness to Zr-MG. Nevertheless, the composition can be continuously changed in a range from Zr_57_Cu_20_Al_10_Ni_8_Ti_5_ (Zr-MG) to at least Zr_20_Cu_71_Al_3.6_Ni_2.9_Ti_1.8_ (Zr-MG 60 wt% Cu). Additional to the compositions in this study, a fully amorphous sample with Zr-MG 20 wt% Cu 10 wt% Ni was produced. Compared to compositions from literature, the adjustable range is large (see Fig. 6) and new BMGs with a significant different chemical composition can be produced. Hence, the HPT can be used to fathom the limits of possible BMG’s chemical compositions.

In summary, it is shown that BMGCs can be produced via HPT and their microstructure and mechanical properties can be adjusted by changing composition and the applied strain. The BMGCs can be forced to mix and the solubility range is highly extended, producing new BMGs. The chemical composition of this new BMGs can be adjusted over a wider range compared to conventional casting and by adding additional elements (e.g. Cu and Ni), the possibility of feasible BMG compositions increases drastically. Changing the composition influences also the mechanical properties and the hardness can be increased by approximately 15%. While the generation of new BMGs is important, even more interesting is the potential for tuning the properties of metal-BMG composites with the possibility to vary the second phase. The second phase can be freely changed in choosing different materials and compositions and its dimensions can be altered from the µm to the nanometer regime.

## Methods

The metallic glass powder (Zr_57_Cu_20_Al_10_Ni_8_Ti_5_, spherical particle with diameters between 1 µm to 40 µm) was fabricated via high pressure gas atomization and was then mixed with crystalline Cu powder (spherical particle with diameters between 10 µm to 40 µm) in the respective compositions and blended by hand. The powder mixture was then filled into the gap between two grooved anvils and compacted by applying 4 GPa and 10° rotation in the HPT. For further deformation, the pressure was increased to 8 GPa (or 9 GPa) and samples with 2 to 500 rotations at room temperature were produced. The dimensions of the specimens were 6 mm in diameter and a height of approximately 450–600 µm. The applied shear strain *γ* can be estimated with

1 $$\gamma =2\pi rN/t$$

where *r* is the radius, *N* is the numbers of rotations and *t* is the thickness after deformation. As the dimensions were approximately the same for all samples, the degree of deformation is adjusted by the numbers of applied rotations (more turns lead to higher deformation) and at the radius where the sample was investigated (the applied strain increases with the radial distance from the center of the disk). For SEM and hardness measurement, the coin-like HPT samples were cut in half, ground and polished to investigate the cross sections. In SEM the back-scatter detector was used to provide mass contrast so that the two phases are more easily distinguishable. Vickers hardness was measured along the diameter on the cross section with a load of 0.5 kg. An error of 2% for the measured hardness values is assumed and the standard deviation errors of the fitted curve were calculated and used for the error bars for Fig. 5. For nanoindentation testing of the powder and the deformed sample, a platform nanoindenter G200 (Keysight Tec) was used and the experiments were conducted under constant indentation strain rate (0.05 s^−1^) to a maximum indentation depth of 500 nm. The hardness was measured continuously over indentation depth and was averaged between an indentation depth between 400 to 450 nm. The non-deformed powder was embedded and then mechanical ground and polished like the surface of the deformed sample. Several particles were indented and for both materials, a mean value and the standard deviation were calculated from all indentations. A 5-circle X-ray diffractometer equipped with a source for Cu-K_α_ radiation was used for XRD phase analysis of the specimens. Half samples were used and the surface was grounded to remove any impurities from the HPT process. The investigated 2-Theta range was concentrated around the first amorphous peak (the strongest).

## References

[1] Gao HL, Shen Y, Xu J (2011). Weibull analysis of fracture strength for Zr55Ti2Co28Al15 bulk metallic glass: Tension-compression asymmetry and porosity effect. J. Mater. Res..

[2] Ketov, S. V. & Greer, A. L. Influence of cyclic loading on the onset of failure in a Zr-based bulk metallic glass. 6716–6721, doi:10.1007/s10853-014-8276-2 (2014).

[3] Meng F, Tsuchiya K, Seiichiro I, Yokoyama Y (2012). Reversible transition of deformation mode by structural rejuvenation and relaxation in bulk metallic glass. Appl. Phys. Lett..

[4] Wang XD (2011). Atomic-level structural modifications induced by severe plastic shear deformation in bulk metallic glasses. Scr. Mater..

[5] Eckert J, Das J, Pauly S, Duhamel C (2007). Mechanical properties of bulk metallic glasses and composites. J. Mater. Res..

[6] Cheng JL (2010). Correlation of the microstructure and mechanical properties of Zr-based *in-situ* bulk metallic glass matrix composites. Intermetallics.

[7] Hays CC, Kim CP, Johnson WL (2000). Microstructure controlled shear band pattern formation and enhanced plasticity of bulk metallic glasses containing *in situ* formed ductile phase dendrite dispersions. Phys. Rev. Lett..

[8] Heilmaier M (2001). Deformation behavior of Zr-based metallic glasses. J. Mater. Process. Tech..

[9] Qiao JC, Pelletier JM (2011). Crystallization kinetics in Cu46Zr45Al7Y2 bulk metallic glass by differential scanning calorimetry (DSC). J. Non. Cryst. Solids.

[10] Wu Y (2011). Formation of Cu-Zr-Al bulk metallic glass composites with improved tensile properties. Acta Mater..

[11] Ott RT, Fan C, Li J, Hufnagel TC (2003). Structure and properties of Zr-Ta-Cu-Ni-Al bulk metallic glasses and metallic glass matrix composites. In Journal of Non-Crystalline Solids.

[12] Deng ST (2011). Metallic glass fiber-reinforced Zr-based bulk metallic glass. Scr. Mater..

[13] Shakur Shahabi H, Scudino S, Kühn U, Eckert J (2014). Metallic glass-steel composite with improved compressive plasticity. Mater. Des..

[14] Conner RD, Dandliker RB, Johnson WL (1998). Mechanical properties of tungsten and steel fiber reinforced Zr41.25Ti13.75Cu12.5Ni10Be22.5 metallic glass matrix composites. Acta Mater..

[15] Liu T, Shen P, Qiu F, Yin Z, Lin Q (2009). Synthesis and mechanical properties of TiC-reinforced Cu-based bulk metallic glass composites. Scr. Mater..

[16] Choi-Yim H, Busch R, Köster U, Johnson WL (1999). Synthesis and characterization of particulate reinforced Zr57Nb5Al10Cu15.4Ni12.6 bulk metallic glass composites. Acta Mater..

[17] Xu YK, Ma H, Xu J, Ma E (2005). Mg-based bulk metallic glass composites with plasticity and gigapascal strength. Acta Mater..

[18] Wang K (2008). Interface structure and properties of a brass-reinforced Ni59Zr20Ti16Si2Sn3 bulk metallic glass composite. Acta Mater..

[19] Lee JG, Park SS, Lee DG, Lee S, Kim NJ (2004). Mechanical property and fracture behavior of strip cast Zr-base BMG alloy containing crystalline phase. Intermetallics.

[20] Wang H, Sen WJY, Liu YT (2016). Effect of the volume fraction of the *ex-situ* reinforced Ta additions on the microstructure and properties of laser-welded Zr-based bulk metallic glass composites. Intermetallics.

[21] Wang DJ, Wei XS, Shen J (2013). Diamond reinforced Al-based bulk metallic glassy composites with improved plasticity fabricated by cold hydro-mechanical pressing. J. Alloys Compd..

[22] Kelly JP (2016). Designing *in situ* and *ex situ* bulk metallic glass composites via spark plasma sintering in the super cooled liquid state. Mater. Des..

[23] Perrière L, Champion Y (2012). Phases distribution dependent strength in metallic glass – aluminium composites prepared by spark plasma sintering. Mater. Sci. Eng. A.

[24] Valiev, R. Z., Islamgaliev, R. K. & Alexandrov, I. V. *Bulk nanostructured materials from severe plastic deformation*. *Progress in Materials Science* **45**, (2000).

[25] Estrin Y, Vinogradov A (2013). Extreme grain refinement by severe plastic deformation: A wealth of challenging science. Acta Mater..

[26] Kormout KS, Yang B, Pippan R (2015). Deformation Behavior and Microstructural Evolution of Cu-Ag Alloys Processed by High-Pressure Torsion. Adv. Eng. Mater..

[27] Bachmaier A, Keckes J, Kormout KS, Pippan R (2014). Supersaturation in Ag-Ni alloy by two-step high-pressure torsion processing. Philos. Mag. Lett..

[28] Kormout, K. S., Pippan, R. & Bachmaier, A. Deformation-Induced Supersaturation in Immiscible Material Systems during High-Pressure Torsion. *Adv*. *Eng*. *Mater*. 1–19, doi:10.1002/adem.201600675 (2016).

[29] Edalati K (2015). Plastic Deformation of BaTiO _3_ Ceramics by High-pressure Torsion and Changes in Phase Transformations, Optical and Dielectric Properties. Mater. Res. Lett..

[30] Razavi-Khosroshahi H, Edalati K, Arita M, Horita Z, Fuji M (2016). Plastic strain and grain size effect on high-pressure phase transformations in nanostructured TiO2 ceramics. Scr. Mater..

[31] Wang YB (2012). Introducing a strain-hardening capability to improve the ductility of bulk metallic glasses via severe plastic deformation. Acta Mater..

[32] Joo S (2015). Work-Hardening Induced Tensile Ductility of Bulk Metallic Glasses via High-Pressure Torsion. Sci. Rep..

[33] Bünz J (2014). Low temperature heat capacity of a severely deformed metallic glass. Phys. Rev. Lett..

[34] Das J (2005). {“Work-Hardenable”} Ductile Bulk Metallic Glass. Phys. Rev. Lett..

[35] Asgharzadeh, H., Joo, S. H., Lee, J. K. & Kim, H. S. Consolidation of Cu-based amorphous alloy powders by high-pressure torsion. *Journal of Materials Science*, doi:10.1007/s10853-015-8877-4 (2015).

[36] Sort J (2004). Cold-consolidation of ball-milled Fe-based amorphous ribbons by high pressure torsion. Scr. Mater..

[37] Sun YF, Nakamura T, Todaka Y, Umemoto M, Tsuji N (2009). Fabrication of CuZr(Al) bulk metallic glasses by high pressure torsion. Intermetallics.

[38] Sun YF, Fujii H, Tsuji N, Todaka Y, Umemoto M (2010). Fabrication of ZrAlNiCu bulk metallic glass composites containing pure copper particles by high-pressure torsion. J. Alloys Compd..

[39] Fogagnolo JB, Oliveira MF, de, Kiminami CS, Bolfarini C, Botta WJ (2004). Consolidation of Easy Glass Former Zr55Cu30Al10Ni5 Alloy Ribbons by Severe Plastic Deformation. J. Metastable Nanocrystalline Mater..

[40] Sauvage X (2014). Structure and properties of a nanoscaled composition modulated metallic glass. J. Mater. Sci..

[41] Ŕv́sz Á, Hóbor S, Lábár JL, Zhilyaev AP, Kovács Z (2006). Partial amorphization of a Cu-Zr-Ti alloy by high pressure torsion. J. Appl. Phys..

[42] Yavari AR, Filho WJB, Rodrigues CAD, Cardoso C, Valiev RZ (2002). Nanostructured bulk Al90Fe5Nd5 prepared by cold consolidation of gas atomised powder using severe plastic deformation. Scr. Mater..

[43] Kündig AA, Schweizer T, Schafler E, Löffler JF (2007). Metallic glass/polymer composites by co-processing at similar viscosities. Scr. Mater..

[44] Edalati K, Yokoyama Y, Horita Z (2010). High-Pressure Torsion of Machining Chips and Bulk Discs of Amorphous Zr50Cu30Al10Ni10. Mater. Trans..

[45] Krämer L, Kormout K, Setman D, Champion Y, Pippan R (2015). Production of Bulk Metallic Glasses by Severe Plastic Deformation. Metals (Basel)..

[46] Tenwick. Enhanced strength in high conductivity Cu alloys. *Mater*. *Sci*. **98**, 543–546 (1988).

[47] Jiang QK (2008). Zr-(Cu,Ag)-Al bulk metallic glasses. Acta Mater..

[48] Men H, Fu J, Ma C, Pang S, Zhang T (2007). Bulk glass formation in ternary Cu-Zr-Ti system. J. Univ. Sci. Technol. Beijing Miner. Metall. Mater. (Eng Ed).

[49] Wang Q (2004). Composition optimization of the Cu-based Cu-Zr-Al alloys. Intermetallics.

[50] Wang YL, Xu J (2008). Ti (Zr)-Cu-Ni bulk metallic glasses with optimal glass-forming ability and their compressive properties. Metall. Mater. Trans. A Phys. Metall. Mater. Sci..

[51] Chen W, Wang Y, Qiang J, Dong C (2003). Bulk metallic glasses in the Zr-Al-Ni-Cu system. Acta Mater..

[52] He Q, Xu J (2012). Locating Malleable Bulk Metallic Glasses in Zr–Ti–Cu–Al Alloys with Calorimetric Glass Transition Temperature as an Indicator. J. Mater. Sci. Technol..

[53] Wiest, A. *et al*. Zr–Ti-based Be-bearing glasses optimized for high thermal stability and thermoplastic formability. **56**, 2625–2630 (2008).

[54] Umetsu, R. Y., Tu, R. & Goto, T. Thermal and Electrical Transport Properties of Zr-Based Bulk Metallic Glassy Alloys with High Glass-Forming Ability. **53**, 2–6 (2012).

[55] Xu Y, Zhang Y, Li J, Hahn H (2010). Enhanced thermal stability and hardness of Zr46Cu39.2Ag7.8Al7 bulk metallic glass with Fe addition. Mater. Sci. Eng. A.

[56] Caron A, Wunderlich R, Gu L, Fecht HJ (2011). Structurally enhanced anelasticity in Zr-based bulk metallic glasses. Scr. Mater..

[57] Kim CP (2009). Fracture toughness study of new Zr-based Be-bearing bulk metallic glasses. Scr. Mater..

[58] Liu JW, Cao QP, Chen LY, Wang XD, Jiang JZ (2010). Shear band evolution and hardness change in cold-rolled bulk metallic glasses. Acta Mater..

[59] Liu XJ (2011). Atomic packing symmetry in the metallic liquid and glass states. Acta Mater..

[60] Ma D (2007). Nearest-neighbor coordination and chemical ordering in multicomponent bulk metallic glasses. Appl. Phys. Lett..

